# Supplementary material for: Ex Vivo Optimization of Donor Lungs with Inhaled Sevoflurane during Normothermic Ex Vivo Lung Perfusion (VITALISE): A Pilot and Feasibility Study in Sheep
Source: Int J Mol Sci. 2024 Feb 19;25(4):2413. doi: 10.3390/ijms25042413 (PMC10888671; doi:10.3390/ijms25042413)
Supplement: Supplementary file 1 [file ijms-25-02413-s001.zip › Supplemenary Material.pdf]

Ex VIvo opTimisAtion of donor Lungs with Inhaled SEvoflurane during normothermic ex vivo lung perfusion (VITALISE): A pilot and feasibility study in sheep.

Timo Steinkühler, BSc; Shuqi Yang, MD; Michiel A. Hu, MD; Jayant S. Jainandunsing, MD; Dr. Neeltina M. Jager, MD PhD; Dr. Michiel E. Erasmus, MD PhD; Prof. Dr. Michel M. R. F. Struys, MD PhD, FRCA; Dr. Dirk J. Bosch, MD PhD; Dr. Matijs van Meurs, MD PhD; Prof. Dr. Matthieu Jabaudon, MD PhD; Dr. Damien Richard, PharmD PhD; Prof. Dr. Wim Timens, MD PhD; Prof. Henri G. D. Leuvenink, MSc PhD; Dr. Gertrude J. Nieuwenhuijs-Moeke, MD PhD

Supplementary Material

Table of Contents

Supplementary Figures **2**

Supplementary Tables **4**

References **6**

Supplementary Figures

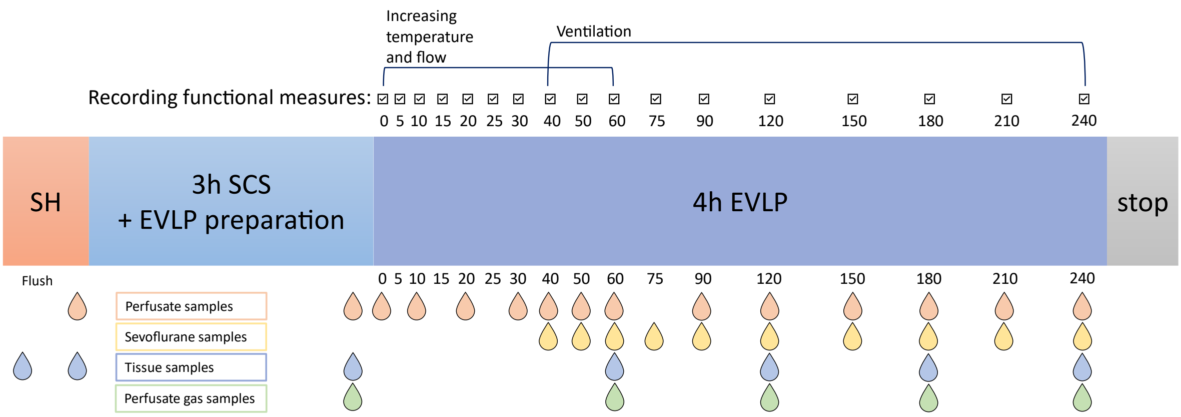

Figure S1 - EVLP sampling timeline. Abbreviations: SH, Slaughterhouse; SCS, static cold storage

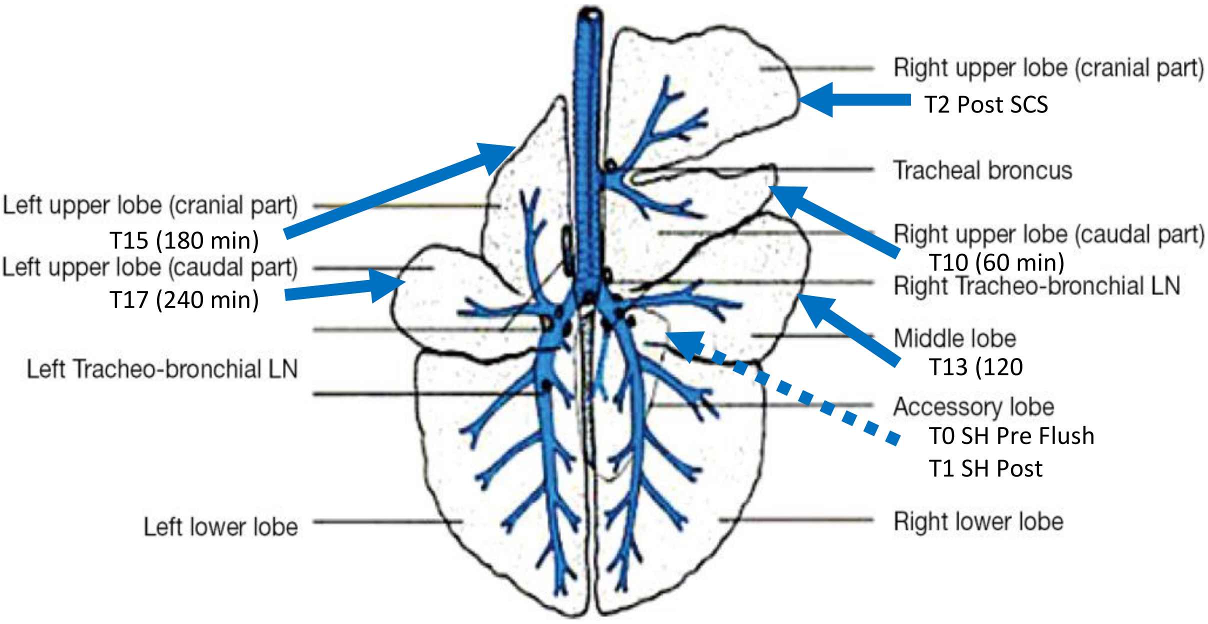

Figure S2 - Biopsy sites.[1] Abbreviations: SCS, static cold storage; min, minutes after start of EVLP; SH, Slaughterhouse

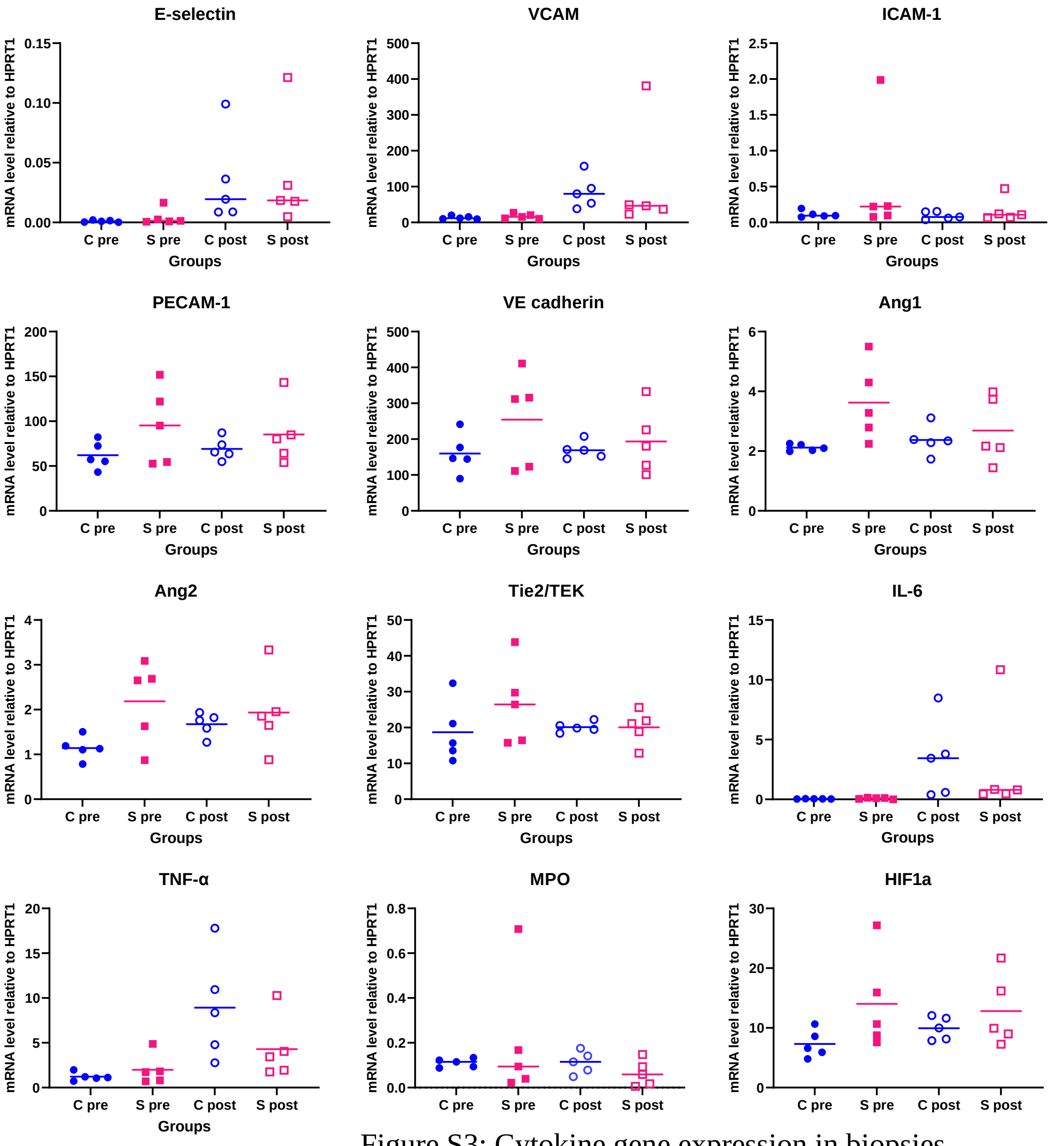

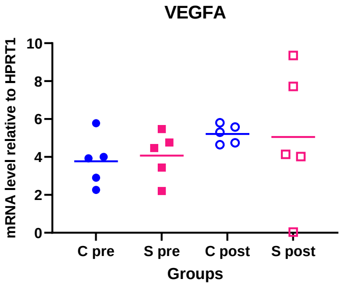

Figure S3: Cytokine gene expression in biopsies.
Abbreviations: Hprt1, Hypoxanthine phosphoribosyltransferase 1; VCAM, Vascular cell adhesion molecule; ICAM-1, Intercellular adhesion molecule 1; PECAM-1, platelet and endothelial cell adhesion molecule 1; VE cadherin, Vascular Endothelial-Cadherin; Ang1, Angiopoietin 1; Ang2, Angiopoietin 2; Tie2/TEK, TEK receptor tyrosine kinase; IL-6, Interleukin 6; TNF-α, Tumor Necrosis Factor Alpha; MPO, Myeloperoxidase; HIF1a, Hypoxia inducible factor 1 subunit alpha; VEGF-a, Vascular Endothelial Growth Factor A

Supplementary Tables

Table S1 - Composition of homemade flush and perfusion solutions

Ingredient	Flush solution	Perfusion solution
NaCL (g/L)	8	2,16
KCl (g/L)	0,4	0,34
MgSO4 (g/L)	0,098	
Na2HPO4 (g/L)	0,046	
KH2PO4 (g/L)	0,063	
D-glucose monohydrate (g/L)	1	
Dextran 40 (g/L)	50	5
Human Albumin (g/L)		70
Glucose anhydrous (g/L)		1,8
NaH2PO4 monobasic (g/L)		0,146
CaCl2 dihydrate (g/L)		0,22
MgCl2 (g/L)		0,11
NaHCO3 (g/L)		1,26
NaOH (2M)		titrate to pH 7,4
**Medications**		
Heparin (IU/L)	5,000	5,000
Dexmethasone (mg/L)		50
Cefuroxime (mg/L)		750
Nitroglycerine (mg/L)	4	6,5
TRIS Buffer (mmol/L)	0,09	

Table S2 - Sample Timepoints

Sample	Timepoint	Sevoflurane	Perfusate	Biopsies	Blood Gas
T0	SH pre Flush			x	
T1	SH post Flush		x	x	
T2	pre EVLP / post SCS			x (+mRNA)	x
T3	Retro Perfusion		x		
T4	0 min (start EVLP)		x		
T5	10 min		x		
T6	20 min		x		
T7	30 min		x		
T8	40 min	x	x		
T9	50 min	x	x		
T10	60 min	x	x	x	x
T11	75 min	x			
T12	90 min	x	x		

T13	120 min	x	x	x	x
T14	150 min	x	x		
T15	180 min	x	x	x	x
T16	210 min	x	x		
T17	240 min	x	x	x (+mRNA)	x
**Total (per exp)**		10	15	7	5
Abbreviations: SH, Slaughterhouse; SCS, static cold storage; Retro, retrograde; min, minutes after start of EVLP; exp, experiment					

Table S3 - Primer/probe sets used for mRNA expression analyses

**Gene**	**Assay ID**	**Encoded protein**
HPRT1	Oa04825272_gH	Hypoxanthine phosphoribosyltransferase 1
E-selectin	Oa04658914_m1	E-selectin
VCAM	Oa04918369_sH	Vascular cell adhesion molecule
ICAM-1	Oa04658649_m1	Intercellular adhesion molecule 1
PECAM-1	Oa04677168_m1	Platelet and endothelial cell adhesion molecule 1
VE cadherin	Oa04661728_g1	Cadherin 5, Vascular endothelial cadherin
Ang1	Oa04876148_m1	Angiopoietin 1
Ang2	Oa04857533_m1	Angiopoietin 2
Tie2/TEK	Oa04724201_m1	TEK receptor tyrosine kinase, Tie2
IL-6	Oa04656315_m1	Interleukin 6
TNF-α	Oa04656867_g1	Tumor Necrosis Factor Alpha
MPO	Oa04654413_g1	Myeloperoxidase
HIF1a	Oa04877335_m1	Hypoxia inducible factor 1 subunit alpha
VEGFA	Oa04653812_m1	Vascular Endothelial Growth Factor A

Table S4 - Amount of sevoflurane administered in mL/min

**Timepoint (min)**	**Sevoflurane administration (mL/h)**
40	5.0 (4.63/5.38)
50	4.0 (3.63/5.65)
60	3.3 (2.63/5.00)
75	3.4 (2.90/3.73)
90	2.8 (2.70/3.35)
120	2.7 (2.45/3.25)
150	2.6 (2.55/3.30)
180	2.8 (2.55/2.90)
210	2.8 (2.35/2.85)
240	2.8 (2.45/3.00)

Data are presented as median (interquartile range).
Abbreviations: min, minutes after start of EVLP

References

1. de la Torre M, Gonzalez-Rivas D, Fernández-Prado R, Delgado M, Fieira EM, Centeno A. Uniportal video-assisted thoracoscopic lobectomy in the animal model. *J Thorac Dis*. 2014;6(Suppl 6):656. doi: 10.3978/j.issn.2072-1439.2014.10.03 [doi].
